# Electrical Stimulation of the Human Cerebral Cortex by Extracranial Muscle Activity: Effect Quantification With Intracranial EEG and FEM Simulations

**DOI:** 10.1109/TBME.2016.2570743

**Published:** 2016-07-19

**Authors:** Lukas Dominique Josef Fiederer, Jacob Lahr, Johannes Vorwerk, Felix Lucka, Ad Aertsen, Carsten Hermann Wolters, Andreas Schulze-Bonhage, Tonio Ball

**Affiliations:** Intracranial EEG and Brain Imaging Lab, Epilepsy Center, Medical Center – University of Freiburg, Freiburg, Germany, the Neurobiology and Biophysics, Faculty of Biology, University of Freiburg, Freiburg Germany, the BrainLinks-BrainTools Cluster of Excellence, University of Freiburg, Freiburg, Germany, and with the Bernstein Freiburg Center, Freiburg; Intracranial EEG and Brain Imaging Lab, Epilepsy Center, Medical Center – University of Freiburg, Freiburg, Germany, and also with the Department of Psychiatry and Psychotherapy as well as Freiburg Brain Imaging, Medical Center – University of Freiburg, Freiburg, Germany; Institute for Biomagnetism and Biosignalanalysis, University of Münster, Münster, Germany, and also with the Scientific Computing and Imaging (SCI) Institute, University of Utah, Salt Lake City, UT-84112, USA; Center for Medical Image Computing, University College London, London, England and with the Institute for Biomagnetism and Biosignalanalysis, University of Münster, Münster, Germany; Neurobiology and Biophysics, Faculty of Biology, University of Freiburg and also with the Bernstein Center Freiburg; Epilepsy Center, Medical Center–University of Freiburg, Freiburg, Germany, the BrainLinks-BrainTools Cluster of Excellence, University of Freiburg, Freiburg, Germany and also with the Bernstein Center Freiburg, Freiburg, Germany; Intracranial EEG and Brain Imaging Lab, Epilepsy Center, Medical Center – University of Freiburg, Freiburg, Germany, the BrainLinks-BrainTools Cluster of Excellence, University of Freiburg, and also with the Bernstein Center Freiburg

**Keywords:** Brain stimulation, electrical stimulation, electrocorticography, electroencephalography, electromyography, endogenous stimulation, finite element analysis, volume conductor head modeling

## Abstract

**Objective:**

Electric fields (EF) of approx. 0.2 V/m have been shown to be sufficiently strong to both modulate neuronal activity in the cerebral cortex and have measurable effects on cognitive performance. We hypothesized that the EF caused by the electrical activity of extracranial muscles during natural chewing may reach similar strength in the cerebral cortex and hence might act as an endogenous modality of brain stimulation. Here, we present first steps toward validating this hypothesis.

**Methods:**

Using a realistic volume conductor head model of an epilepsy patient having undergone intracranial electrode placement and utilizing simultaneous intracranial and extracranial electrical recordings during chewing, we derive predictions about the chewing-related cortical EF strength to be expected in healthy individuals.

**Results:**

We find that in the region of the temporal poles, the expected EF strength may reach amplitudes in the order of 0.1–1 V/m.

**Conclusion:**

The cortical EF caused by natural chewing could be large enough to modulate ongoing neural activity in the cerebral cortex and influence cognitive performance.

**Significance:**

The present study lends first support for the assumption that extracranial muscle activity might represent an endogenous source of electrical brain stimulation. This offers a new potential explanation for the puzzling effects of gum chewing on cognition, which have been repeatedly reported in the literature.

## I. Introduction

Endogenous modulation of neuronal activity through ephaptic coupling at the cellular level has received increasing attention during the last years. Multiple groups could show that the local electric fields (EF) generated by active neurons feed back onto themselves [1]–[15]. This ephaptic coupling is especially effective for naturalistic EF [12]. EF strength in the order of magnitude of 0.2 V/m may be sufficient to elicit these effects [13]. Transcranial electric stimulation (TES) also influences the EF of the brain [16] and has been shown to have an impact on diverse brain functions [17]–[24], including working memory and learning, at similar cortical EF strength as in the endogenous case [21], [25].

Besides neuronal activity, electrical muscle activity is another source of endogenous EF [26], [27]. Particular strong muscle activity close to the brain occurs during chewing. Interestingly, using different batteries of cognitive tests, it was shown that cognitive performance is enhanced for 15–20 min after gum chewing [28]. Chewing during the cognitive testing itself significantly reduced test performance [28]. These findings were previously explained by indirect effects, such as unspecific psychological arousal induced by the chewing activity. Here, we consider the alternative hypothesis that the cognitive effects of gum chewing are at least in part a direct consequence of cortical electrical endogenous stimulation caused by the electrical activity of muscles during mastication.

However, assessing the cortical EF caused by muscle activity to be expected in healthy individuals is a challenging task. It is not possible to directly measure the intracranial signal generated by extracranial muscles in healthy individuals, as this would require implanted electrodes. Therefore, to derive quantitative predictions on the extent of such signals, we proceeded in the following steps: first, we utilized the unique opportunity offered by patients with diagnostically implanted electrodes where it is possible to simultaneously measure both intra- and extracranial electrical signals. These measurements were obtained during chewing of typical soft hospital food. Next, we addressed the problem that the results from these patient measurements cannot be directly transferred to the case of healthy individuals, as in the former but not the latter the skull is breached by craniotomy defects as a consequence of the surgical electrode implantation. Such skull defects can have a substantial impact on volume conduction that has to be taken into account. To do so, here we used detailed finite element method (FEM) volume conductor head modeling calibrated with the patient data to estimate the strength of effects to be expected in the absence of craniotomy defects, by closing the skull defects in the otherwise identical FEM model. Finally we performed an experiment to determine the range of electromyogram (EMG) strength during chewing of food with a range of consistencies, including chewing gum. In summary, by this procedure we arrived at quantitative predictions on the strength of chewing-related (ChR) cortical EF to be expected in healthy individuals.

Our results show that particularly in the region of the temporal poles, which are geometrically close to the masticatory muscles, the strength of ChR cortical EF to be expected in healthy individuals may well reach relevant levels that could modulate cortical activity and have functional consequences. Thus, our findings lend first support to the assumption that extracranial muscles can act as endogenous brain stimulators.

## II. Methods

### A. Intracranial ChR Potentials During Weak Chewing

#### 1) Patients

Five patients under evaluation for neurosurgical treatment of medically intractable epilepsy were included in the present study (see Table I). Electrodes were implanted subdurally for a period of 5–10 days, depending on the individual clinical requirements, to localize seizure onset zones and determine eloquent brain areas to be preserved during surgical intervention, such as those responsible for language functions and motor control. The electrode contacts were stainless steel or platinum discs 4 mm in diameter, mounted on a flexible silicone substrate (Ad-Tech, Racine, WI, USA) at a 10-mm center-to-center interelectrode distance. Most patients had additional linearly arranged strip electrodes or penetrating depth electrodes in the hippocampus (1-mm diameter, ten contacts with a 5-mm contact-to-contact distance), though the effects in the depth electrodes were not of the object of the present study. The type and placement of all electrodes were solely determined by the requirements of preneurosurgical diagnostics. All patients provided written informed consent prior to the study.

#### 2) Data Acquisition

Electrocorticogram (ECoG) and electroencephalogram (EEG) (standard 10–20 positions [29] as far as allowed by the wounds) were simultaneously recorded at a sampling rate of 1024 Hz, with a high-pass filter of 1 Hz and a low-pass filter of 344 Hz, using a clinical AC EEG-system (IT-Med, Usingen, Germany). Digital video, synchronized with neural data, was recorded at 25 frames per second at VGA resolution. Channels with technical recording problems (e.g., broken wires) were excluded from further analyses.

#### 3) Trial Selection

Trials were acquired during natural food intake of the patients without any prior instruction. Chewing events were marked manually within interictal time periods based both on the digital video recordings and on the typical, pronounced ChR EMG bursts of the masticatory muscles visible in the EEG (e.g., in channels T4 and F8). The EMG onset and end were marked for each chewing event [c.f. Fig. 2(a), for an example], and their arithmetic mean was defined as the 0-s time point for each trial. In this way, a total of 1652 trials were acquired from five patients (S1: 551 trials; S2: 438 trials; S3: 252 trials; S4: 264 trials; S5: 147 trials).

#### 4) Analysis

The ECoG data were separately re-referenced to a common average reference (CAR), as it is common in ECoG studies [30]–[32]. The EEG data were re-referenced to Cz, as the clinical environment did not allow for a clean CAR reference and Cz was least susceptible. Trials were excerpted from the continuous data from –2 to 2 s with respect to the 0-s time point in the chewing event. In this time window, sliding-window fast Fourier transformations were performed with a window length of 250 ms and a step width of 24.41 ms (corresponding to 256 and 25 sampling points, respectively). A baseline period was defined in a pre-event time window (200 ms) selected around the center between consecutive chewing events [see Fig. 2(b)]. The relative time–frequency spectra were divided by the median baseline power averaged across trials and then scaled logarithmically. A two-tailed sign test was employed for statistical analysis, and correction for multiple testing was performed following the false discovery rate (FDR) approach suitable for correlated *p*-values (as for neighboring time and frequency bins), with a q-level of 0.001 [33].

To compare intra- and extracranial ChR EMG amplitudes, we high-pass filtered the data at 100 Hz and, for each chew event, calculated the ChR EMG amplitude as the difference between the 10th and 90th percentile in a 100-ms time window around the center of each trial. To test the influence of these parameters on the results, we also performed the analysis with 55 Hz high-passed data, extracted peak-to-peak amplitudes, and varied the window length from 50 to 300 ms.

### B. Volume Conductor Modeling

#### 1) FEM Head Models

**Fig. 1:**
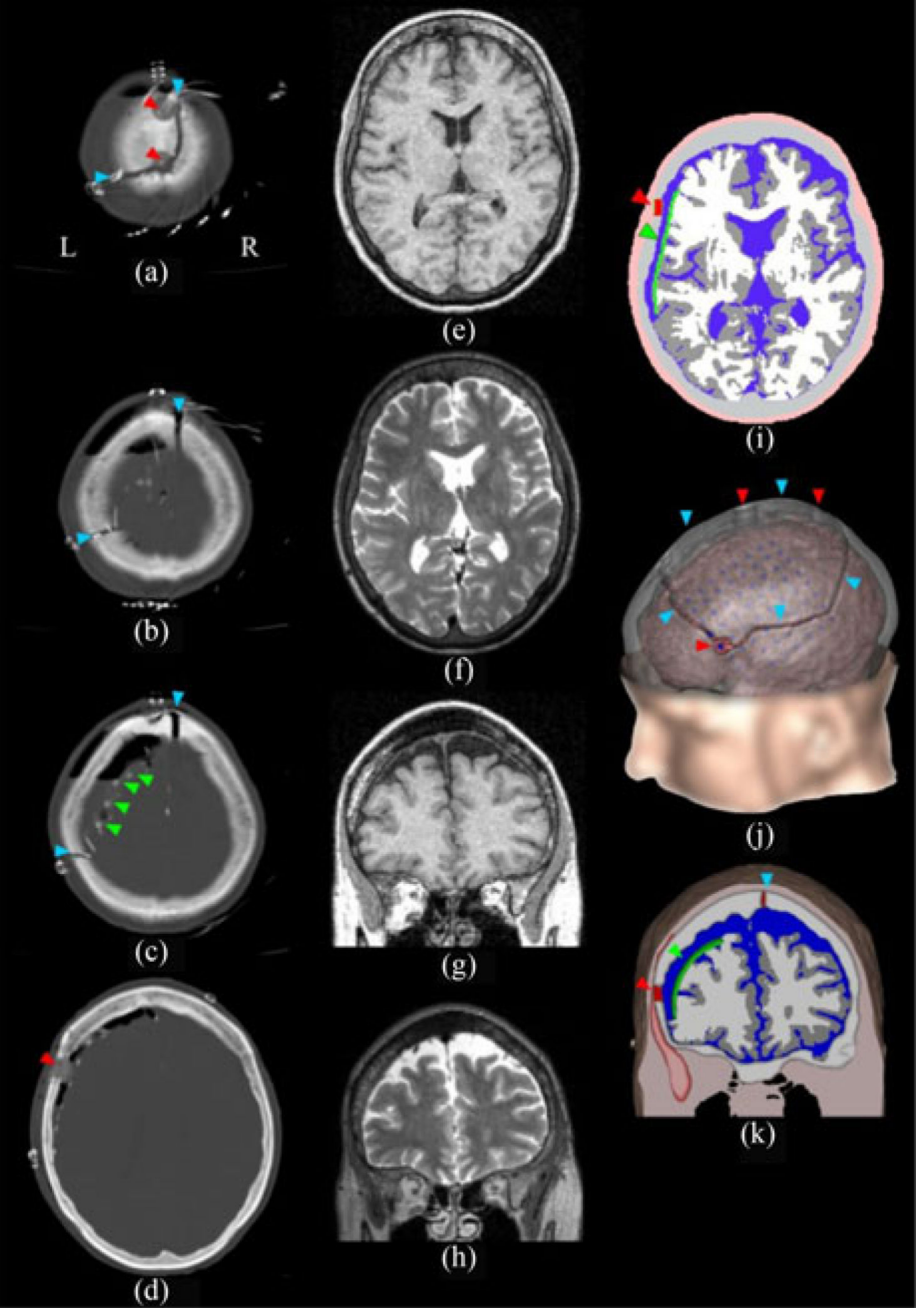
CT and MRI imaging data and volume conductor head model. **(a)–(d)** Axial CT images taken after subdural electrode implantation. **(e) and (f)** Axial slices through preoperative T1 and T2 weighted MRI data, respectively. **(g) and (h)** Coronal slices through preoperative T1 and T2 weighted MRI data, respectively. **(i)** Axial slice through segmented data of Head Model 1 (HM 1, with craniotomy defects and with grid, see Section II). For comparison with the MRI data the slice was taken at the same position as in (e) and (f). Soft tissue: light pink; air: black; temporalis muscle: dark pink; skull: light gray; craniotomy defects: red; ECoG grid: green; CSF: blue; gray matter: dark gray; and white matter: light gray. **(j)** 3-D visualization of HM 1. Gray matter surface: pink; electrodes: blue; skull: transparent gray. **(k)** 3-D coronal slice through volume conductor model (HM1). For comparison with the MRI data, the slice was taken at the same position as in (g) and (h). Conventions as in (i). The red, turquoise, and green arrowheads indicate the burr holes, saw lines, and the electrode grid, respectively.

A volume conductor head model of patient S3 was used to model the extra- to intracranial conduction of electric potentials caused by dipolar sources located in the left temporal muscle. Patient S3 was chosen because here we had the best imaging data for building the FEM model. Additional control simulations were performed using head models adapted to the burr hole configuration of the other patients (S1, 2, 4, and 5). Whole-head MRI volumes were acquired before surgery in a Siemens Vision scanner at 1.5 T using a T1 MPRAGE sequence and in a Siemens TrioTrim using a T2 SPC sequence, both at a 1-mm isotropic resolution [see Fig. 1(e)–(h)]. The head model was created using the brain extraction tool [34] and the FMRIB Automated Segmentation Tool [35] provided by the FMRIB Software Library toolbox [36]. It included white matter, gray matter, cerebrospinal fluid (CSF), skull, and soft tissue. Anatomically unrealistic segmentation outcomes were corrected manually.

The model was then extended semiautomatically to include facial soft tissue and internal air. The left temporal muscle was manually segmented based on the T1 and T2 data. The positions of burr holes, saw lines, and of the electrode grid were determined based on the postimplantation T1 MRI and CT scans. Because iatrogen air cavities and metal artifacts, made coregistration and segmentation unreliable, the craniotomy defects were included in the following way. Burr holes in the skull model were created by calculating the position of cylinders (12-and 16-mm diameters, determined from CT) around the burr hole centers and by replacing the skull tissue within the cylinder volume by CSF. The saw lines were generated based on path nodes set on a surface mesh of the outer skull surface. The connection line of these points was then projected onto a mesh of the inner skull surface. All skull points between these trajectories were replaced by CSF.

The sphenoidal and oval foramina have clinical relevance as they act as high-conductance tunnels, facilitating the recording of brain signals [37]–[40]. Moreover, multiple studies report on the importance of skull foramina in conducting epileptic spikes to the scalp surface [37], [41]–[44]. Therefore, we assumed that these foramina could also play a role in the opposite direction, facilitating the propagation of EMG potentials to the brain, particularly in the case of the pterygoid masticatory muscles, which are very close to some major foramina. Hence, we manually added (bilaterally) the following foramina of the skull base to the model: the foramen ovale, rotundum and spinosum, the fissure orbitalis superior, and the carotid canals. Foramina and fissures were modeled as cylinders filled with white matter or blood as anatomically appropriate, and with diameters of 1–7 mm, based on [45]. Carotid canals were manually segmented from the MRI data using Seg3D (Seg3D Development Team).

Due to substantial swellings and shifts of brain tissue following surgery, as well as due to iatrogen air cavities and metal artifacts, an automatic coregistration of the electrode grid (determined in postimplantation 3-D images) to the preoperative MRI used for the volume conductor model was not reliable. Thus, the position of the electrode contacts was reconstructed on the 3-D surface taking into account the positional information from the postimplantation MRIs, CT, and a lateral 2-D X-ray image. The main challenge in constructing the grid model was to adapt it to the local gyral geometry constrained by the physical properties of the grid. This was achieved by the following steps.

1) Creating a triangulated hull around the brain that followed the outer brain surface but not the individual gyri. To this end, we used the “mesh_shrinkwrap” algorithm (Bioelectromagnetism MATLAB Toolbox, [46]).
2) Selecting the corners of the electrode grid on the hull, based on the CT, X-ray, and MRI data.
3) Extracting a 3-D patch defined by the corners from the hull.
4) Projecting the 3-D patch coordinates into 2D using the isomap algorithm (MATLAB Toolbox for Dimensionality Reduction, [47]). This algorithm was especially suitable for this task as it is designed to well preserve the geodesic distances between neighboring data points [47].
5) Finding the closest three neighbors within the 2-D patch of each electrode center.
Finally, transforming the resulting 2-D triangulation back into a 3-D triangulation using the original 3-D coordinates of the patch.

This created an accurate representation of the electrodes within the ECoG grid, molded onto the surface of the cortex, while respecting electrode array geometry and in the correct position as verified using postimplantation imaging data. As, in FEM simulations, contacts over edges or corners lead to current leakage, special care was taken to ensure that the reconstructed grid was “sealed” by face-to-face contacts, thus preserving the grid’s insulating properties. Geometry-adapted hexahedral meshes were generated based on the segmented images with Vgrid [48] and visualizations were performed using SCIRun (freely available from the SCIRun Development Team). Every 3-D surface visualized using SCIRun was smoothed using the default settings of the “FairMesh” module. Based on the procedures described above, three different head models were created:

Head Model 1 (HM 1): The complete head model with burr holes, saw lines and the insulating grid; Head Model 2 (HM 2): identical to HM 1, but without burr holes and saw lines, to model the effects of the insulating ECoG grid separately; Head Model 3 (HM3): identical to HM 1, but without both the craniotomy defects and the insulating electrode grid, thus, representing the situation in healthy individuals.

#### 2) FEM Simulations and Source Models

FEM forward calculations were computed with SimBio [49] using the St. Venant dipole modeling approach [50], [51]. The conductivity values used were derived from the resistivity values used in [52], namely white matter 0.14 S/m, gray matter 0.33 S/m, CSF 1.54 S/m, blood 0.63 S/m, skull 0.0063 S/m, muscle 0.11 S/m, soft tissue 0.17 S/m, and internal air 0.002 S/m. Foramina filled with both blood and nerves were modeled with 0.38 S/m, which is the average of blood and white matter conductivities. Burr holes and saw lines, as determined from CT data, were filled with CSF. For the insulating silicone ECoG grid a conductivity of 1e–45 S/m was used, which is the numerical conductivity closest to 0 S/m that SimBio could model.

We used the following source models to represent the electrical activity of the chewing muscles: Source Model 1 (SM 1): a single dipole central in the belly of the temporal muscle (i.e., the muscle contributing most force to jaw closure in chewing); Source Model 2 (SM 2): to account for the thin, superior part of the temporal muscle, seven dipoles were placed within the belly of the temporal muscle and one dipole in the superior part in front of a burr hole; Source Model 3 (SM 3): to investigate the impact of the pterygoid muscles, in particular of the medial pterygoid which also contributes significant force to jaw closing in chewing and which is situated adjacent to major skull foramina (e.g., the foramen ovale), a dipole was placed in the medial pterygoid muscles in front of the formaen ovale.

### C. Noninvasive ChR Potential Measurements and Analysis

The intracranial data of the present study were acquired while patients ate the typically soft hospital food (soup, cake, etc.) that is served to patients after having undergone major head surgery, during which a partial incision of the temporal muscle is likely. Thus, the ChR EMG amplitudes were relatively low and largest contralateraly to the side of surgery. To characterize EMG amplitudes that can be expected during both weak and strong chewing in the general healthy individuals population, measurements were conducted on three healthy participants (P1–P3) under the following six conditions:

1) eating yoghurt with mashed banana (referred to as Yoghurt);
2) eating banana;
3) eating a raw carrot;
4) chewing gum;
5) eating a mouthful of hard-to-chew gummi candies; and
6) eating a mouthful of licorice.

EMG potentials were recorded from 128 standard electrode positions in the 10–5 system [53], with Cz as reference electrode. As in the patients, the amplitude of the chewing events was determined as the potential difference between the 10th and 90th percentile of the EMG signal in the 100-Hz high-pass filtered data in a time window of 100-ms duration around EMG maximum. Across participants and conditions 1639 chew events were analyzed. For each participant, the median amplitude of each channel across the chewing events of one condition was calculated. Then the median amplitude over channels was calculated for each participant. The mean chewing amplitude over the three participants was calculated for each condition and used as noninvasive scaling data (cf. below).

### D. Cortical EF Analysis

To estimate the single-trial cortical EF that can be expected in healthy individuals during weak to strong chewing, we proceeded in the following steps.

**Fig. 2:**
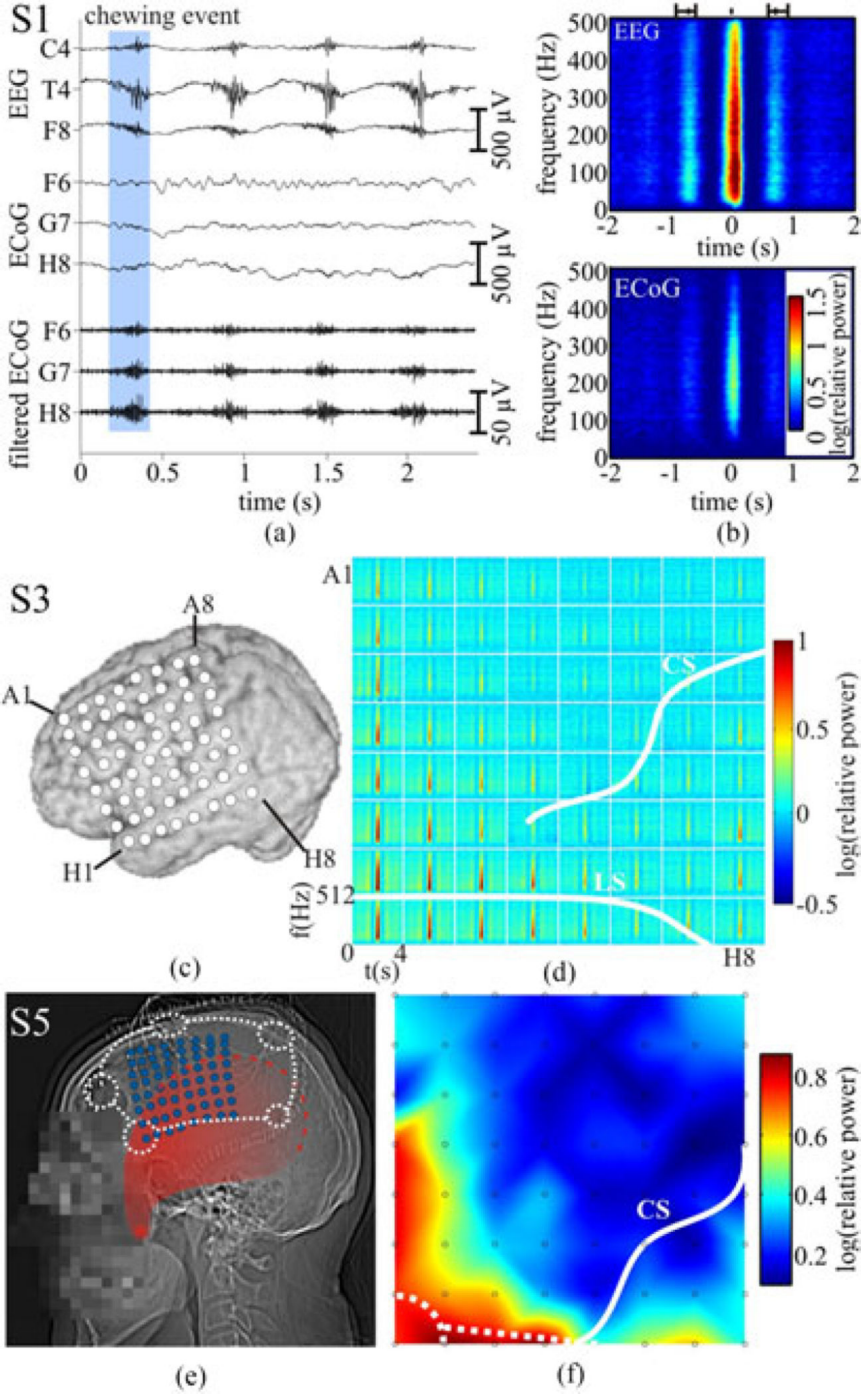
Chewing–related (ChR) EEG and ECoG data recorded in patients. **(a)** Ongoing EEG from channels C4, T4, and F8 of S1 together with the data from three ECoG channels (F6, G7, and H8) simultaneously recorded in the same patient. The time epoch of a chewing event, as marked for the analysis, is indicated by a blue box. The EEG traces reveal distinct EMG bursts, and close inspection of the ECoG channel H8 also reveals ChR high-frequency bursts, albeit of much lower amplitude than in EEG. The three lower traces show the high-pass-filtered ECoG signal from the same channels, enhancing the visibility of ChR high-frequency bursts. **(b)** Time-resolved ChR relative spectral power changes in the EEG channel T4 and ECoG channel H8 involving a broad frequency range. Median time points of the preceding and following chewing event are indicated above the time–frequency plot (error bars: interquartile range). Color encodes the logarithmic power change relative to the baseline (see Section II for further details). **(c)** ECoG grid position in relation to the brain surface obtained from patient S3’s MRI data. **(d)** Time–frequency spectra of ChR responses. The course of the lateral sulcus (LC) and the central sulcus (CS) are depicted by white lines. Note the spatially widespread distribution bridging the LS. **(e)** Patient S5: Lateral X-ray with superimposed positions of implanted electrodes (blue), burr holes (white dashed discs), saw lines (white dashed lines), and the temporal muscle (red) with the temporal line (red dashed line) as its origin and the coronoid process of the mandibular bone (red asterisk) as its insertion. The variation in transparency reflects the thickness of the temporal muscle, which increases toward the coronoid process. **(f)** Intracranial topography of chewing–related events (ChREs) in the gamma frequency range (32–400 Hz). Electrode positions are marked with black circles. The saw lines and burr holes are indicated by white dashed lines and discs.

**Fig. 3:**
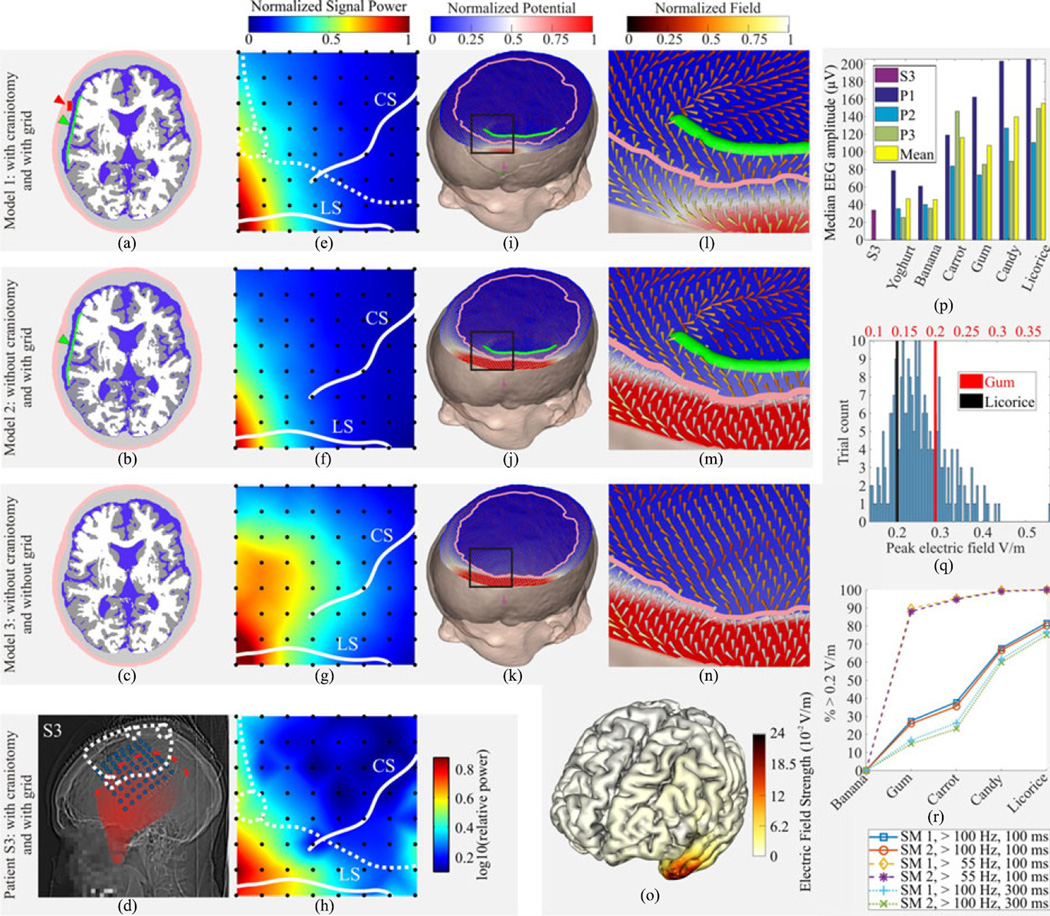
FEM simulation compared to intracranial recordings. **(a)–(c)** Axial slices through all three head models. Craniotomy (red) and silicone grid (green) indicated by red and green arrows, respectively. Soft tissue: light pink; skull: light gray; CSF: blue; gray matter: dark gray; and white matter: light gray. **(d)** Lateral X-ray with superimposed positions of implanted electrodes (blue), burr holes (white dashed discs), saw lines (white dashed lines), and the temporal muscle (red) with the temporal line (red dashed line) as its origin, and the coronoid process of the mandibular bone (red asterisk) as its insertion, the variation in transparency reflects the thickness of the temporal muscle that increases toward the coronoid process. **(e)–(g)** Interpolated EMG power caused by SM 2 reproducing the power maxima in the anterio-inferior corner of the grid as observed in the recorded ECoG data (h). Electrode positions are marked with black disks. The saw lines and burr holes are indicated by white dashed lines and discs, and the lateral (LS) and central sulci (CS) are indicated by continuous white lines. **(h)** Intracranial topography of ChRE in the gamma frequency range (32–400 Hz). Conventions as in (e)–(g). **(i)–(k)** Skin: beige, skull: dark gray; dipole: magenta; ECoG grid: green. Outline of inner skull surface is marked by pink line (interrupted at the positions of the saw lines in HM 1). **(l)–(n)** Magnifications of the regions indicated by black boxes in (i)–(k) showing both the normalized potential (the background colors using a blue–white–red color scale) and the normalized EF (foreground cones using a red–yellow–white color scale) around the edge of the silicone grid. **(o)** Cortical EF (median across trials) expected in healthy individuals during chewing of licorice using SM 1. Maximal EF strength was found in the temporal pole and anterior medial and lateral temporal cortex. **(p)** ChR median EEG amplitudes of patient S3 and, for each chewing condition, of participants P1–3. **(q)** Distribution of the peak cortical EF strength across trials expected in healthy individuals for gum chewing (red units) and licorice (black units) chewing. All trials to the right of the red and black bars exceeded 0.2 V/m. Previous studies suggest modulatory effects on ongoing brain activity above this threshold (see Section IV). **(r)** Percentage of trials producing peak cortical EF exceeding 0.2 V/m for each chewing condition (yoghurt not shown as the percentage was always 0%), Source Models (SM) 1 and 2 (SM 3 not shown as always 100%) and different analysis parameters for high-pass frequency and amplitude window.

1) We determined the strength of the current dipole(s) in the masticatory muscles that would be required to generate ECoG potentials of the same amplitude as measured in the intracranial calibration data, i.e., in the individual chewing events of S3. To model the potential reversal expected in an amplitude, the simulated ECoG potentials were multiplied by a factor of 2 before matching them to the calibration data. These simulations were based on HM 1, i.e., with craniotomy skull defects (burr holes, saw lines) and the insulating electrode grid [see Figs. 1(e), (f) and 2(a)]. This first step gave us the distribution of current dipole(s) strength needed to generate the data measured in S3.
2) Then, we computed the single-trial cortical EF resulting from the current dipole(s) derived in step 1, but using HM 3 without craniotomy and grid [see Fig. 3(c)]. The cortical EF was computed for the whole extent of the cerebral cortex and the amplitude and positions of the EF maxima were determined. This second step gave us the distribution of the peak cortical EF strength expected in a healthy individual (without craniotomy and electrode grid).
3) To calculate the EF to be expected during chewing with different muscle strength, the single-trial EF strength values determined in step 2 were scaled by the ratio of the amplitude of the noninvasive scaling data to the median EEG ChR amplitude of each trial [see Fig. 3(p)]. This third step gave us the single-trial distribution of the peak cortical EF expected in healthy individuals during a variety of chewing conditions [e.g., Fig. 3(q)].
4) Finally, we determined the percentage of trials with peak EF exceeding 0.2 V/m [see Fig. 3(r)].

This analysis was carried out with SMs 1, 2, and 3.

## III. Results

### A. Chewing-Related Events (ChREs) are Clearly Present in the ECoG

Examples of simultaneously recorded ChREs in EEG and ECoG from S1 are shown in Fig. 2(a). Consistent with our expectations, ChR bursts of high-frequency activity were clearly visible in the ongoing EEG recordings in all patients (shown here for S1) from all temporal and fronto-lateral channels [e.g., T4 and F8 in Fig. 2(a)]. However, similar high-amplitude ChREs were never observed in the simultaneously recorded ECoG [Fig. 2(a), middle three traces]. Nevertheless, high-frequency ChR bursts with peak-to-peak amplitudes of approx. 30 µV became visible in the ongoing ECoG after high-pass filtering [cutoff = 100 Hz, Fig. 2(a), three bottom traces]. In the unfiltered ECoG traces, close inspection revealed relatively low-amplitude ChR bursts [Fig. 2(a), highlighted by blue boxes] in the ongoing (not trial-averaged) recordings at some ECoG channels.

The time–frequency power spectra of the EEG data typically showed a very pronounced broadband ChR power increase in the frequency range up to the Nyquist frequency of the recordings [max. ca. 500 Hz, Fig. 2(b)], which is typical of EMG activity. ChR ECoG spectra showed a similar time–frequency pattern, but with amplitudes smaller by about one order of magnitude [Fig. 2(b)], which is typical for extra- to intracranial propagation. Significant ChREs-induced power increases (*p* < 0.001, FDR-corrected) could be observed in 406 of the 410 (99%) analyzed ECoG contacts, including grid and strip electrodes in the five patients investigated (all electrodes in S1, S3, and S4, and all but two electrodes in S2 and S5).

### B. The Topography of Intracranial ChREs

ChREs spectral power modulations revealed a spatially widespread distribution over the grid array [Fig. 2(d)]. Also, the maximal power was found in the anterolateral corner of the grid, intermediate power at other positions close to the edge of the grid, and the smallest power increases in the center of the grid. These widespread effects extended without any interruption over the anatomical borders of the lateral sulcus (LS) and central sulcus (CS), and were not focalized to electrodes with oro-facial responses elicited by electrical cortical stimulation.

### C. The Intracranial ChR Power Topography is Reproduced by FEM Volume Conductor Modeling

The basic power topography of intracranial ChREs [see Figs. 2(f) and 3(h)], with most power in the anterior–inferior corner of the grid could be well reproduced by an FEM forward simulation, based on dipole sources in the belly and in the thin superior part of the temporal muscle (SMs 1 and 2), including both skull defects (burr holes, saw lines) and the insulating ECoG grid [HM 1, Figs. 1(e), (f) and 2(a)]. EF vectors were forced around the edges of the silicone substrate [see Fig. 3(i)–(k), (l)–(n)]. This basic topography was also reproduced with the other control simulations (SM 3 and burr holes adapted to other patients, see Section II).

### D. The Silicone Grid has a Strong Shielding Effect

Comparing the results of HM 1 with those of HM 2 [cf. Fig. 3(e), (f)], it could be seen that the craniotomy defects have only a small impact on intracranial EMG power topography and power amplitudes (accounting for power amplitude differences of only approx. 6%). However, comparing the results of HMs 1 [see Fig. 3(e), (i), (l)] and 2 [see Fig. 3(f), (j), (m)] with those of HM 3 [see Fig. 3(g), (k), (n)], it became apparent that the insulating ECoG grid has a strong shielding effect. Its removal accounts for an ~27% increase of intracranial EMG power (referring to the peak EMG power across the grid). This is best illustrated in Fig. 3(l), (m), where one can see how EF vectors are forced to run parallel to the ECoG grid, and in Fig. 3(g), with substantially increased EMG power in the head model without ECoG grid.

### E. EEG and ECoG Amplitudes of ChREs

The median ChRE amplitude was determined across EEG and ECoG channels for all patients and subjects using the difference between the 10th and 90th percentile in a 100-ms window relative to the center of the events. For S1–5 median EEG amplitudes were 24.9, 25.1, 33.7, 38.8, and 29.4µV, respectively, with. 30.4 µV mean. Median ECoG amplitudes were 5.0, 5.4, 6.4, 4.3, and 7.6 µV, respectively, with 5.7 µV mean. Thus median chewing event amplitudes were attenuated by a factor of 5.0, 4.6, 5.3, 9.0, and 3.9, respectively, with 5.5 mean, from EEG to ECoG.

Mean ChR EEG amplitudes across healthy participants in the different chewing conditions were yoghurt 46.6 µV, banana 45.7 µV, raw carrot 116.4 µV, gum 107.3 µV, candy 139.9 µV, and licorice 155.2 µV. Results are summarized in–Fig. 3(p).

### F. Cortical EF Expected in Healthy Individuals

The strongest EF were, irrespective of Head and Source Model, located at the temporal pole [see Fig. 3(o)]. For the EF expected in healthy individuals, depending on the source model and chewing condition, the percentage of chewing events generating EF strengths above 0.2 V/m varied from 0 to 100%. The predicted gum-ChR EF strengths were above 0.2 V/m in 27.5% of trials in SM 1 (one dipole in the belly of the temporalis muscle), 25.9% in SM 2 (seven dipoles in the belly of the temporalis muscle and one in the superior part), and 100% in SM 3 (one dipole in the medial pterygoid muscle in front of the foramen ovale). For details relating to the other conditions, as well as parameter variations, cf. Fig. 3(r). Median chewing repetition rate ranged, across all patients, participants, and conditions, from 0.82 to 1.8 Hz.

## IV. DISCUSSION

### A. ChREs Mainly Arise From EMG Activity

For a number of reasons, it appears most plausible to assume that the ChREs observed in the present study, for the most part, arise from the EMG activity of the masticatory muscles, rather than result from neural activity related to sensory processing or motor control of the act of chewing. In two previous functional magnetic resonance imaging (fMRI) studies, BOLD signal changes related to chewing, tongue tapping, or swallowing [54], [55] were found focally in regions of the primary sensory and motor cortex with a spatial response pattern clearly different from the spatially widespread distribution of ChREs in our study, which extended smoothly over functional and structural boundaries [see Fig. 2(d)]. Furthermore, the spectral profile of the ChREs showed broadband frequency increases instead of the typical of event-related neural population responses of the cortex with both low-frequency suppression and gamma-band increases [56], [57]. Nevertheless, since previous fMRI studies have shown a cortical involvement in the motor control of chewing (see above), the presence of a small, focal neural signal component masked by the high-amplitude extracranial EMG seems likely, although nonexperimentally performed chewing might produce much less cortical involvement than its experimental counterpart. Further work will be necessary to isolate this presumably weak neural signal component, if possible at all.

### B. FEM Modeling Predictions of ChR Cortical EF in Heathy Subjects

From our FEM simulations based on three head models as summarized in Fig. 3, it follows that high-amplitude extra-to-intracranial signal conduction should also take place in healthy individuals with an intact skull. This assumption was tested through volume conductor modeling determining the amplitudes of signals resulting from extra-to-intracranial EMG propagation if craniotomy defects and insulating silicone grid were removed from the head model, while keeping all other factors constant (see Fig. 3). Not surprisingly, craniotomy defects facilitated extra-to-intracranial EMG propagation and hence their removal from the head model slightly reduced the amplitudes of the EMG signals that reach the brain [compare Fig. 3(e) and (f)]. However, when additionally removing the insulating ECoG grid [see Fig. 3(g)], it became evident that the grid acts as a strong electrical shield and that removal of the grid therefore leads to substantially increased intracranial EMG amplitudes. The signal gain by removal of the insulator outweighs the signal loss by closing the craniotomy, resulting in a net signal increase in the “healthy” head model (HM 3) as compared to HM 1 with craniotomy and with grid [see Fig. 3(e), (g)]. This effect was observed consistently in a range of control simulations with source configurations with different levels of spatial detail. These results also imply that, in the opposite direction, cortical potentials generated below the ECoG grid should be attenuated in EEG recordings above the insulating grid, even in the presence of craniotomy defects as indeed shown by [58]–[60] (however, see also [61], [62]). The assumption that signals in the gamma-frequency range, in which EMG has high amplitudes, can indeed overcome the intact skull is further supported by earlier studies showing that, in the other direction, task-related gamma responses originating from the brain can be detected in scalp EEG in healthy individuals [63], [64].

### C. Could Cortical EF Induced by Chewing Modulate Brain Activity?

#### 1) Cortical EF Induced by Chewing Are in the Proper Amplitude Range to Modulate Brain Activity

Recent evidence suggests that even weak EF (in the range of 0.2 V/m) can have a direct influence on the activity of neocortical neural networks [13]. While low-amplitude EF did not trigger additional action potentials, they did induce substantial shifts in the timing of action potentials [12]–[14]. Neuronal networks have been shown to be even more sensitive to EF than single neurons [15]. The theoretical sensitivity limit of elongated neurons was calculated to be in the order of 0.01 V/m [65] but no empirical study has yet confirmed this prediction.

Typical stimulation intensities used in previous transcranial random noise stimulation (tRNS) studies were in the 1-mA peak-to-peak amplitude range [20], but already 0.4 mA tRNS has been shown to modulate cortical function [21]. The maximal cortical EF strength directly beneath a stimulation pad and at 1 mA was found to be 0.45 V/m [25], hence 0.18 V/m EF can be expected to be responsible for the effects observed with 0.4 mA tRNS, which matches well the threshold of 0.2 V/m determined empirically by Reato and colleagues [13]. The assumption that cortical EF in this order of magnitude has a modulatory effect on neuronal network function is strongly supported by data from recent *in vitro* experiments [12], [13].

With our SM 2 (seven dipoles in the belly of the temporalis muscle and one in the superior part), 25.9% of chewing events scaled for gum chewing in healthy individuals produced peak EF strengths larger than the empiric threshold of 0.2 V/m. When varying the window length used to calculate the ChR amplitudes from 50 to 300 ms, this percentage ranged from 33.1% to 14.7%, respectively [cf. Fig. 3(r) for more details]. As we gradually increased the firmness of the chewed food the proportion of chewing events above 0.2 V/m also increased: carrot 35.5%, candy 66.5%, and licorice 80.1%. These strong EFs involved the temporal poles, extending to the medial and lateral anterior temporal regions [see Fig. 3(o)]. SM1 (one dipole in the belly of the temporalis muscle) produced slightly larger values as SM 2 while SM 3 (one dipole in the medial pterygoid muscle in front of the foramen ovale) continuously produced EF above 0.2 V/m. These differences are understandable as dipoles in the superior part of the temporalis muscles are in a “good” (spatially close) position to generate potentials measurable in the ECoG grid, but contribute little to the anterior temporal EF, which is mainly caused by dipoles in the belly of the temporal muscle. The opposite is true for dipoles representing activity of the pterygoid masticatory muscles. Due to their position, dipole sources here must be of relatively high amplitudes to generate appreciable ECoG potentials but they can “easily” cause high anterior temporal cortical EF, because they are situated close to the foramina of the skull base, which act as high-conductance tunnels connecting the extracranial and intracranial space [66], [67].

#### 2) Cortical EF Induced by Chewing Are in the Proper Frequency Range to Modulate Brain Activity

tRNS, i.e., brain stimulation with a broadband signal similar to the EMG examined here, is particularly effective in modulating cortical network function. tRNS can improve neuroplasticity underlying motor and perceptual learning with effects lasting at least 60 min after stimulation [19], [20]. The effect of tRNS appeared to depend mainly on the high-frequency (100–600 Hz) component of the stimulation signal, whereas the lower frequencies seem to be less important [20]. In addition to producing lower frequency components in the ECoG, the ChR EMG had pronounced effects in the ECoG in the range from 100 to at least 500 Hz [see Fig. 2(a), (b), (d), (f)]. Moreover, Fröhlich and McCormick [12] presented strong evidence that naturalistic stimulation (using previously recorded ongoing EF) was more effective at entraining network activity than artificial EF modulated by a sine function, likely because the former consisted of sharp rising ramps with high slopes, similar to the time course of EMG activity in our study.

#### 3) Role of Chewing Repetition Rate

Besides the frequency contents of the EMG generated with each individual chewing event, the repetition rate of these events (how fast or slow one chews) may also play a role in our context. Anastasious *et al*. [14] reported that weak EF oscillating at low (<8 Hz) frequencies are particularly effective for entraining action potentials in rat cortical slices. Similarly, Ozen *et al*. [16] demonstrated that TES at 0.8–1.7 Hz significantly entrained neuronal activity in anesthetized and sleeping, but not in behaving, rats. In humans, Marshall *et al*. [17] could show that TES oscillating at 0.75 Hz during non-rapid-eye-movement sleep significantly increased declarative memory retention rates. By contrast, 5-Hz TES did not induce any changes in declarative memory retention rates. Kirov *et al*. [18] could consequently extend the results of Marshall *et al*. to wakefulness, also using 0.75-Hz TES. Across all patients and healthy participants, the median chewing repetition rate ranged from approx. 0.8 to 1.8 Hz. This repetition rate range is further supported by literature [68] and quite close to the stimulation frequencies described above and could thus favor the entrainment of neuronal activity.

#### 4) Cortical EF Induced by Chewing May Modulate Brain Activity and Influence Cognitive Performance

Together, these results show that on the one hand, the cortical EF to be expected in healthy individuals should depend on the exact recruitment pattern of the masticatory muscles. At the same time, though, our findings indicate that the effects to be expected in healthy individuals might be in the same order of magnitude (0.1–1 V/m), frequency range (100–500 Hz), and repetition rate (1–2 Hz) as EF caused by external technical (tRNS) and endogenous neuronal sources that have both been shown to have an impact on neural network activity.

Thus, taking together previous insights that even weak EF have a modulating impact on cortical network dynamics, findings from tRNS stimulation, and our present findings on how endogenous EF propagate to the human cortex during chewing, it appears possible that ChR EMG acts as an endogenous type of brain stimulation, potentially exerting similar effects on brain functions as are elicited by exogenous brain stimulation, in particular tRNS.

### D. Cortical EF Induced by Chewing: A Possible Explanation for Gum Chewing Effects on Cognition

Chewing gum has repeatedly been reported to have effects on cognitive functions [28], [69]–[71]. By administering a battery of cognitive tasks to participants who chewed gum either prior to or during testing, it was recently confirmed that chewing is associated with changes in cognitive performance that are not present in nonchewing controls. Critically, in chewing subjects, a worsening in cognitive performance was observed during chewing, whereas a consecutive enhancement in performance took place when the chewing preceded the cognitive measurements [28]. The beneficial effects of chewing were reported to last for a time period of 15–20 min after the subjects had chewed gum. These effects were previously explained by indirect psychological effects, in particular by unspecific arousal. In contrast, based on the findings of the present study we propose that the observations on cognitive performance may at least partly be explained by direct electrical stimulation of the brain by one’s own EMG. The cortical EF to be expected, especially in the anterior temporal lobe [see Fig. 3(o)], in healthy individuals during gum chewing might be in the same order of magnitude as both exogenously and endogenously caused EF that modulate cortical neuronal function (see above). The temporal pole and the adjacent area of the anterior and medial temporal lobe have been implicated in a wide range of cognitive functions [72]–[74] and (subtle) modulation of neuronal activity in these regions by masticatory EMG may, therefore, indeed contribute to the reported cognitive effects of chewing.

Generally, the underlying mechanisms and hence the range of effects that can be achieved with brain stimulation techniques goes far beyond the consequences of unspecific effects such as arousal [75]. The effects of tRNS have, for example, been linked to the phenomenon of stochastic resonance [20]. The notion of endogenous brain stimulation presents a novel principle by which interfering with and modulation of neural activity in the human brain may be possible. Among the many topics for further research that arise, evaluating the potential of endogenous brain stimulation as a new experimental tool and even for clinical application, complementary to the exogenous, technical brain stimulation currently used exclusively for this purpose, will be of particular importance.

### E. Limitations

Although we took great care to construct a detailed and precise analysis, some limitations of our results need to be discussed.

#### 1) Sample Size and Calibration/Scaling Procedure

The results are based on a small sample, five epilepsy patients, only one of which we used for volume conduction modeling, and three healthy participants, which obviously restrains the generalization of our results. These should therefore be considered as tentative until confirmed in a larger sample. We took great care to use conservative parameters for the calibration and scaling procedure. By using the difference between the 10th and 90th percentile as chewing amplitude, we increased the robustness against noise but likely underestimated the true peak-to-peak amplitude of the chewing events. Progressive pooling of the noninvasive scaling data using the median instead of mean further increased our robustness against outliers but reduced the final percentage of trials above 0.2 V/m by an average of 7.8%. Similarly, by 100 Hz high-passing the ECoG signal before ChR amplitude analysis, the low-frequency components of the muscle activity (30–100 Hz) were discarded, again giving conservative estimates. To illustrate this, we show results of the calibration and scaling procedure for 55 Hz (above line-noise) high-passed data in Fig. 3(r).

#### 2) Source and Head Modeling

Our simple source models qualitatively reproduced the measured intracranial topographies well, but more detailed EMG source models would further approximate the real electrical activity induced by chewing. A more detailed representation of the skull-base chewing muscles would be desirable but could probably further increase the ChR EF.

The head model used in our study is also limited. Due to the brain shift that occurs when the skull of the patient is opened during surgery, the alignment of the preoperative MRI and the postimplantation images was most likely suboptimal. Therefore, we must expect some inaccuracy in our head model. It would be advantageous to use the postimplantation MRI for model construction, but this was hindered by large iatrogen air cavities as well as by large metal artifacts. We see two possibilities to improve our modeling in future work. First, following [76], if postimplantation MRI with inverted phase-encoding direction has additionally been measured, it should be possible to correct postimplantation MRI artifacts using a reversed gradient artifact correction approach [77]. Another strategy could be to model the brain shift as reported by [78] and subsequently use it for an improved registration of a preoperative MRI and a postimplantation CT. This procedure would also make it possible to model the metal contacts of the electrodes that could introduce local EF distortions. As only 4.15% of the silicone grid would be replaced by open metal contacts, we however anticipate that results should be influenced rather minimally.

The conductivities used in our study are widely used, but their accuracy could be further improved, such as by taking into account their inter- and intraindividual variabilities [79]–[81] and frequency dependence [80], [82]. Moreover, we could try to incorporate the known inhomogeneous and anisotropic conductivity of skull and brain [83]–[86]. As shown by [84] and [85], however, brain anisotropy only plays an important role for sources deep in the brain while we investigated sources outside of the brain. Taken together, therefore, we do not expect significant differences in the results for our specific simulation setup, due to these various model simplifications.

## V. CONCLUSION

We presented our first results toward clarifying whether endogenously produced EF beyond those arising from neuronal activity, e.g., in our case ChR EMG, can influence brain activity and function. Using an FEM head model, calibrated with intracranial ECoG data from an epilepsy patient and noninvasive EEG data from healthy participants, we could show that the amplitude of the ChR EMG expected to reach the cortex of healthy individuals during strong chewing might indeed be sufficiently strong to have such effects. The simulated amplitudes of the ChR cortical EF that we found were very close to the stimulation thresholds previously suggested for both endogenous and exogenous brain stimulation [12], [13], [21], [25]. The present study demonstrates that the combination of simultaneous intra- and extracranial EEG recordings with detailed FEM volume conductor modeling is a powerful approach to assess the impact of muscle activity on the human brain. We believe that this approach will also be useful in further studies on the electrical muscle effects on the brain. For example, such future research might gain further insight by using data from intracranial stereotactic EEG recordings alongside ECoG. Stereotactic recordings may offer additional information as such electrodes are sometimes implanted close to the temporal poles and skull base, where according to our findings, muscles effects should be especially strong.

## Figures and Tables

**TABLE I:** Patient Overview

Pat.	Age	Sex	Diagnosis/Lesion	Grid Localization	Seizure Onset
S1	34	f	Temporal-lobe epilepsy (R), FCD	Fronto-parietal (R)	Frontal
S2	50	f	Frontal-lobe epilepsy (R), FCD	Frontal (R)	Frontal
S3	48	f	Fronto-central epilepsy (L), FCD	Frontal (L)	Frontal
S4	21	f	Frontal-lobe epilepsy (R), FCD	Frontal (R)	Frontal
S5	54	m	Epilepsy (L), post-trauma substance defect	Fronto-temporal (L)	Frontal

pat.: patient; m: male, f: female; FCD: focal cortical dysplasia; R: right; L: left, temp.: temporal, med.: medial, ant.: anterior.
